# Predictive value of contrast-enhanced carotid ultrasound features for stroke risk: a systematic review and meta-analysis

**DOI:** 10.3389/fneur.2025.1487850

**Published:** 2025-05-16

**Authors:** Shili Zhou, Pinjing Hui

**Affiliations:** ^1^Department of Stroke Center, The First Affiliated Hospital of Soochow University, Suzhou, China; ^2^Department of Ultrasound, The Second Affiliated Hospital, Hengyang Medical School, University of South China, Hengyang, China

**Keywords:** carotid artery plaque, contrast-enhanced ultrasound, ischemic stroke, carotid stenosis, meta-analysis

## Abstract

**Objective:**

To elucidate the contrast-enhanced ultrasound (CEUS) features of carotid artery plaques in patients who have experienced an ischemic stroke (IS).

**Methods:**

A computerized search was conducted in databases such as Pub-Med, EMSCO, and Ovid to identify studies reporting CEUS findings of carotid artery plaques. Patients were categorized as IS and non-IS based on clinical and radiological diagnosis, and the quantitative and semi-quantitative CEUS data were analyzed for differences between the two groups.

**Results:**

After the computerized search, a total of 13 eligible studies, comprising 3,092 participants (1,953 with stroke), were included for analysis. IS patients exhibited significantly higher plaque enhancement intensity versus control group (SMD = 0.71, 95% CI: 0.32, 1.11). The positive rate of plaque enhancement within the plaques was significantly higher in IS patients versus non-IS patients (OR = 3.25, 95% CI: 1.86, 5.68). The sensitivity of hyperintense lesion-based diagnosis of stroke was 0.68 (95% CI: 0.54, 0.80), and the specificity was 0.61 (95% CI: 0.47, 0.73), with an area under the curve (AUC) of 0.697.

**Conclusion:**

There are significant differences in CEUS characteristics of carotid artery plaques between IS and non-IS patients. IS patients display markedly augmented plaque enhancement intensity and a higher rate of positive enhancement compared to non-stroke individuals. These noteworthy findings have critical implications in enhancing the accuracy of IS diagnosis and improving the stratification of stroke risk for patients.

**Systematic review registration:**

This study is registered with the International Platform of Registered Systematic Review and Meta-analysis Protocols (INPLASY), 202540006.

## 1 Introduction

Ischemic stroke (IS) stands as a prominent worldwide contributor to both mortality and disability, presenting a substantial risk to public health and welfare (1). Atherosclerosis, a persistent inflammatory, metabolic, and multifaceted condition impacting the inner lining of medium and large arteries, emerges as a primary causative factor of IS, with carotid artery atherosclerotic plaque playing a significant role in its development (2). Traditionally, the assessment of stroke risk has focused on the degree of carotid stenosis. While severe carotid artery narrowing remains an important risk factor, emerging evidence suggests that patients with non-severe stenosis may also experience ischemic events (3, 4). Recent studies indicate that the stability of carotid artery atherosclerotic plaque, rather than just the degree of stenosis, is closely linked to the occurrence of ischemic stroke (IS). In fact, 25–50% of IS events are associated with the rupture of vulnerable plaques (5–7).

The distinctive features of unstable carotid artery plaques can be detected and measured using a range of non-invasive imaging techniques, including ultrasonography (US), computed tomography (CT), high-resolution magnetic resonance imaging (MRI), and nuclear imaging methods (8, 9). These advanced imaging modalities provide valuable information beyond just the degree of arterial narrowing, allowing for improved risk stratification and targeted management of patients at risk of ischemic stroke. Ultrasonography is an excellent screening tool for carotid artery atherosclerosis, as it is cost-effective, rapid, and widely accessible, enabling frequent follow-up examinations (10, 11). Contrast-enhanced ultrasound (CEUS) is a novel non-invasive technique that employs contrast agents containing gas microbubbles, which generate strong echo signals under ultrasound, enhancing image contrast and allowing for clearer visualization of tissue perfusion and structural features (12). As a “tracer” of the vascular system, CEUS can clearly delineate the contours of the vascular intima and carotid artery atherosclerotic plaques, including ulcerated plaques, enabling the assessment of plaque stability based on morphological characteristics (13, 14).

To date, however, there has been a paucity of multi-center, large-scale studies investigating the CEUS features of carotid artery plaques in the ischemic stroke population. To address this gap, we evaluate the CEUS characteristics of carotid artery plaques in patients with ischemic stroke.

## 2 Methodology

### 2.1 Literature search strategy

We conducted a literature search in the Embase, PubMed, and Ovid electronic databases to screen studies that reported the CEUS features of carotid artery plaques in patients with IS. The search terms used included “stroke,” “carotid plaque,” and “contrast-enhanced ultrasound.” The search was limited to publications up to June 1, 2024, without any language restrictions. Next, we conducted a thorough manual search through the bibliographies of the chosen articles to uncover any supplementary studies that could bear relevance to the subject matter.

### 2.2 Study selection criteria

The following predefined eligibility criteria were utilized for study inclusion: (1) Participants: Patients diagnosed with carotid artery atherosclerotic plaques were enrolled. All patients underwent CEUS prior to carotid endarterectomy. Based on the North American Symptomatic Endarterectomy Trial criteria (NASCET), the patients were categorized into two groups: those with asymptomatic and those with symptomatic internal carotid artery stenosis attributable to plaques. Symptomatic internal carotid artery stenosis was defined as the onset of neurological manifestations associated with the ipsilateral carotid artery within the preceding 120 - day period. Other potential etiologies of stroke, such as cardioembolism, were strictly excluded. Notably, none of the patients with asymptomatic internal carotid artery stenosis due to plaques had a history of ischemic events resulting from carotid artery stenosis. (2) Intervention: All participants underwent CEUS examination. (3) Outcome measures: Quantitative or semi-quantitative CEUS characteristics of carotid artery plaques, with clinical and imaging diagnoses of IS and non-ischemic stroke (non-IS). (4) Study design: No restrictions on study type. Research with fewer than 10 participants, along with individual case reports and case series, were omitted from consideration. Two separate evaluators screened the collected articles independently, with any disparities being reconciled by a third reviewer.

### 2.3 CEUS plaque enhancement

CEUS plaque enhancement, plaque enhanced intensity was calculated by subtracting baseline from peak intensities in the core, plaque shoulder, and vessel lumen.

### 2.4 Data extraction

A systematic data extraction process was carried out using an Excel spreadsheet to collect the following information from the included studies: publication year, first author, study design, participant count, and outcomes. Two reviewers independently extracted the data and verified the information, with any disagreements addressed with a third reviewer.

### 2.5 Heterogeneity assessment

The heterogeneity across the included studies was evaluated utilizing the corrected *p*-value and the I-squared (I^2^) statistic. Studies were deemed to exhibit negligible heterogeneity when the *I*^2^ statistic was below 50%, prompting the use of a fixed-effects meta-analytic model. Conversely, an *I*^2^ value of 50% or greater was interpreted as indicative of substantial heterogeneity, leading the authors to employ a random-effects approach to provide a more conservative statistical description of the effect sizes.

### 2.6 Statistical analysis

The analysis of the data was conducted utilizing the meta package in the R programming language, and figures were generated accordingly. For quantitative data on plaque enhancement, the pooled effect size was reported as the standardized mean difference (SMD) with its 95% confidence interval (CI). For qualitative data, such as the presence or absence of plaque enhancement or intraplaque neovascularization (IPN), the pooled effect size was represented by the odds ratio (OR) with its 95% CI. Statistical significance was determined by whether the 95% CI of the SMD or OR contained 0 or 1, respectively. The diagnostic accuracy was analyzed using the R package meta4diag.

## 3 Results

### 3.1 Literature screening and selection

Initially, the literature search yielded 425 articles, and after eliminating duplicates, 141 articles remained. Subsequently, a screening of titles and abstracts produced the exclusion of 78 non-clinical studies, resulting in 63 full-text articles for further evaluation of their eligibility. Among these, 14 articles were excluded due to the inability to extract the specified data, 29 articles did not have a stroke control group, and 7 articles focused only on pediatric populations. Ultimately, 13 studies were included in the analysis.

### 3.2 Characteristics of included studies

The 13 included studies involved a total of 3,092 participants, of whom 1,953 had ischemic stroke (IS). The most commonly used contrast agents were SonoVue, Sonazoid, and Optison, with SonoVue being the most frequently employed. The regions of interest (ROI) included the plaque, plaque surface, and plaque shoulder (Table 1).

**Table 1:** Included studies characteristics.

First author	Year	Enrollments	With stroke	Agent	Region of interest
Xiong (28)	2009	71	35	SonoVue	Entire plaque
Saito (29)	2014	50	19	Sonazoid	Entire plaque and shoulders
Luo (30)	2019	116	62	SonoVue	Entire plaque
Jain (31)	2020	60	32	SonoVue	Entire plaque and surface
Li (14)	2023	660	349	SonoVue	Entire plaque
Li (32)	2024	61	32	SonoVue	Entire plaque
Tan (33)	2022	188	72	SonoVue	Entire plaque and surface
Huang (34)	2021	24	161	Optison	Entire plaque
Li (35)	2018	116	62	SonoVue	Entire plaque
Cui (36)	2023	321	162	SonoVue	Entire plaque
Huang (37)	2010	176	81	SonoVue	Entire plaque
Zhao (38)	2022	60	60	SonoVue	Entire plaque
Cui (13)	2022	50	12	SonoVue	Entire plaque

### 3.3 Meta-analysis of quantitative CEUS plaque enhancement

**Figure 1:**
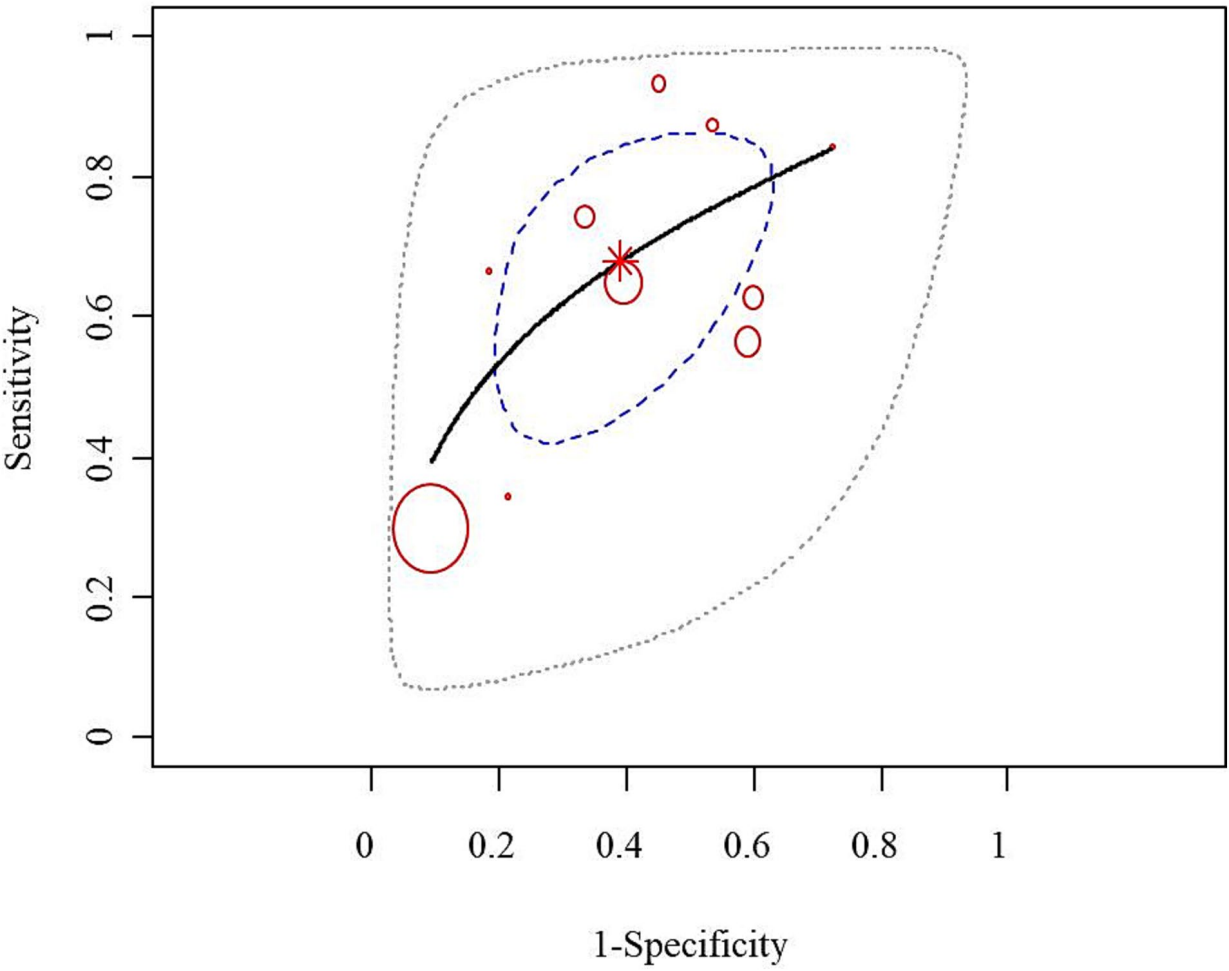
The flow chart for study retrieval and selection.

Three studies reported quantitative analysis of CEUS plaque enhancement, comprising 116 IS patients and 121 non-IS controls. Considerable diversity was evident across the studies, indicating substantial heterogeneity (*I*^2^ = 51%, *p* = 0.13), and a random-effects model was employed. The results demonstrated a higher plaque enhancement intensity in IS patients versus the control group (SMD = 0.71, 95% CI: 0.32, 1.11) (Figure 1).

### 3.4 Diagnostic value of semi-quantitative lesion analysis for stroke

**Figure 2:**
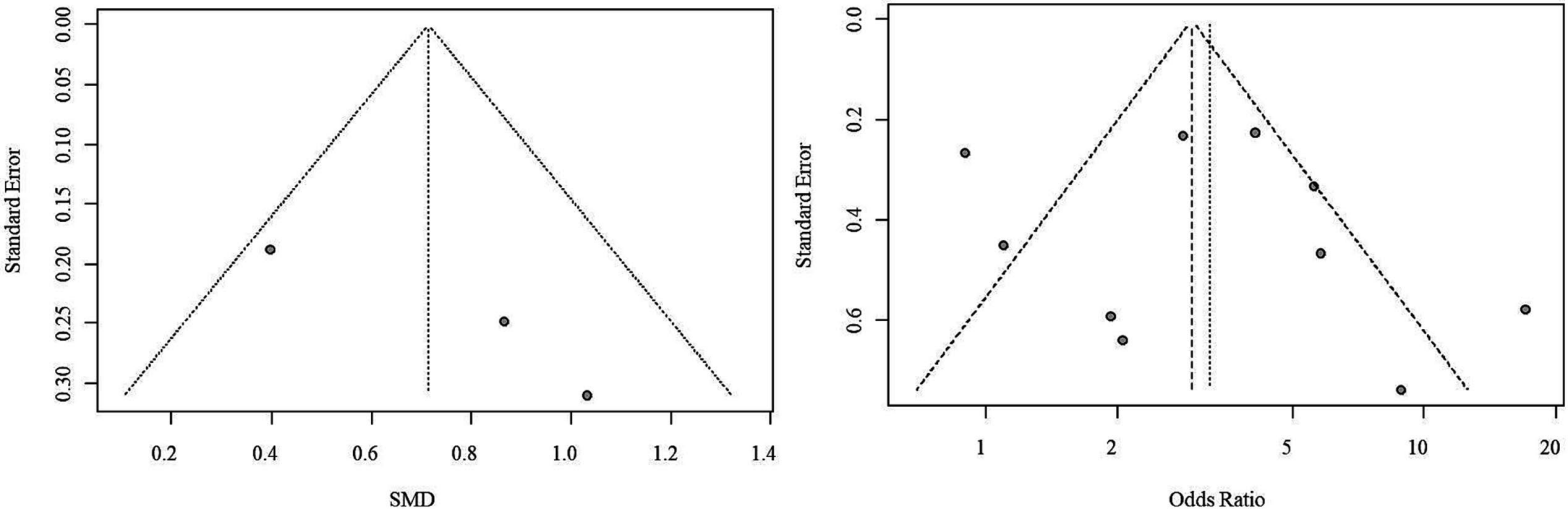
The forest plot of enhanced intensity in plaque by quantitative analysis. Significant variation was apparent among the studies, reflecting considerable heterogeneity (*I*^2^ = 51%, *p* = 0.13), prompting the application of a random-effects model. Findings revealed a notably elevated intensity of plaque enhancement in patients with ischemic stroke compared to the control group (SMD = 0.71, 95% CI: 0.32, 1.11).

For the stroke group, semi-quantitative positivity was considered as true positive (TP), and semi-quantitative negativity as false negative (FN). In the non-stroke group, semi-quantitative negativity was considered as true negative (TN), and semi-quantitative positivity as false positive (FP). A meta-analysis was performed to evaluate the diagnostic value of semi-quantitative positivity for stroke. The SROC scatter plot did not show a clear “shoulder-arm” pattern, and the Spearman correlation coefficient was 0.633 (*p* = 0.076), suggesting no threshold effect. The pooled sensitivity of semi-quantitative positivity for diagnosing stroke was 0.68 (95% CI: 0.54, 0.80), the pooled specificity was 0.61 (95% CI: 0.47, 0.73), and the AUC was 0.697 (Figures 2A,B).

### 3.5 Meta-analysis of semi-quantitative CEUS plaque enhancement

**Figure 3:**
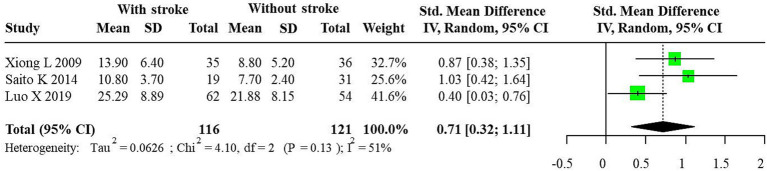
Forest plot of the sensitivity and specificity of semi-quantitative positivity for the diagnosis of stroke. The combined sensitivity of semi-quantitative positivity for stroke diagnosis was 0.68 (95% CI: 0.54, 0.80), while the combined specificity stood at 0.61 (95% CI: 0.47, 0.73), with an AUC of 0.697.

Ten research studies presented semi-quantitative evaluations of plaque enhancement. In this analysis, the absence of enhancement or localized enhancement restricted to the plaque’s edge was deemed as negative, while linear and widespread enhancement were regarded as positive outcomes. The rate of positive plaque enhancement was notably higher in IS when compared to those without IS. Considerable variability was observed among the studies (*I*^2^ = 80%, *p* < 0.001), leading to the adoption of a random-effects model. The findings pointed towards a significantly elevated positive rate of CEUS plaque enhancement in IS versus non-IS patients (OR = 3.25, 95% CI: 1.86, 5.68) (Figure 3).

### 3.6 Publication bias assessment

**Figure 4:**
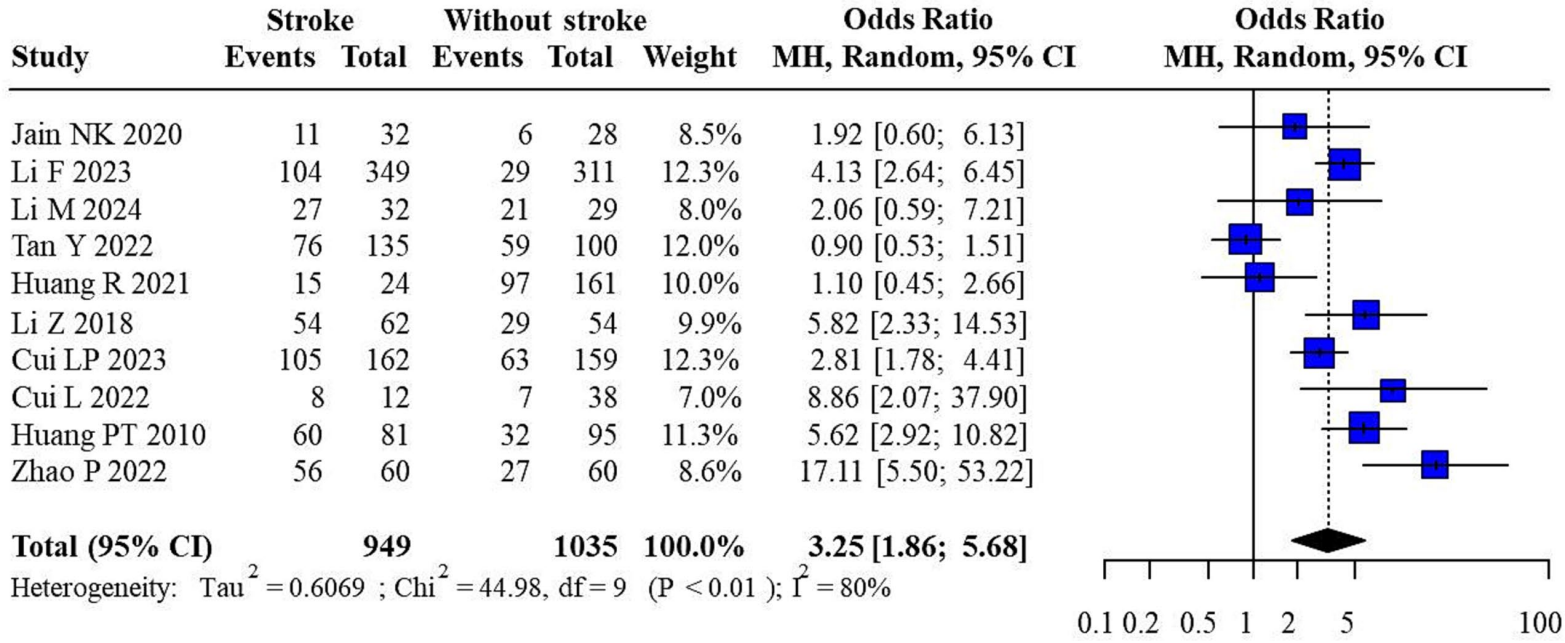
SROC curve of semi-quantitative positivity for the diagnosis of stroke. The scatter plot of the SROC did not exhibit a distinct “shoulder-arm” pattern, and the Spearman correlation coefficient was 0.633 (*p* = 0.076), indicating the absence of a threshold effect.

Due to the limited number of studies available for each outcome, the feasibility of conducting Begg’s test and Egger’s test to assess potential publication bias was restricted. Nonetheless, upon visually inspecting the funnel plots, indications of potential publication bias for both outcome measures were noted (Figure 4).

## 4 Discussion

**Figure 5:**
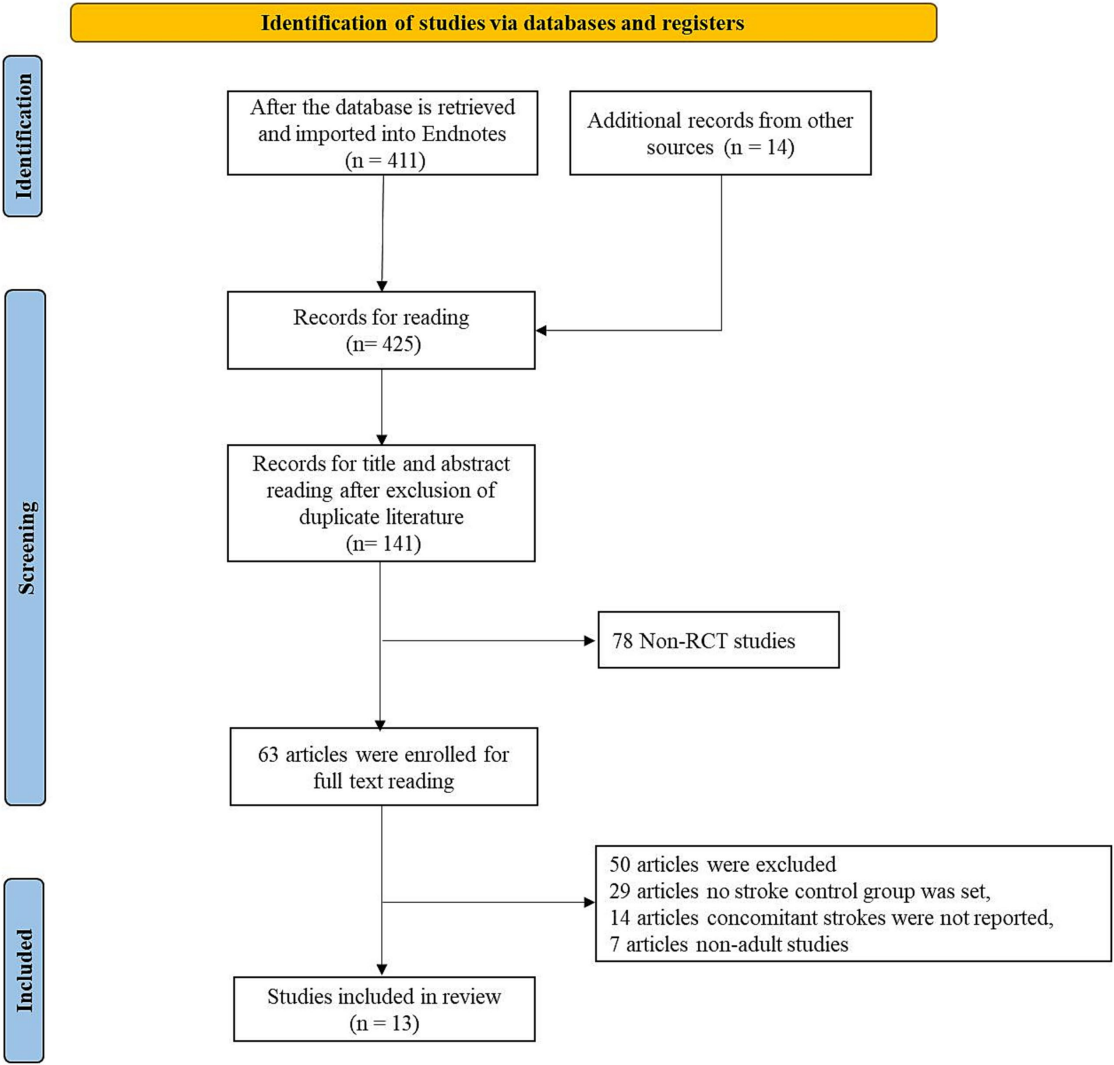
The forest plot of enhanced intensity in plaque by semi-quantitative analysis. Considerable heterogeneity was observed among the studies (*I*^2^ = 80%, *p* < 0.001), leading to the adoption of a random-effects model. The outcomes highlighted a significantly higher rate of positive CEUS plaque enhancement in ischemic stroke patients compared to non-ischemic stroke patients (OR = 3.25, 95% CI: 1.86, 5.68).

CEUS has gained popularity in its application for quantifying the structural characteristics of carotid atherosclerotic plaques, evaluating plaque stability, and assessing neovascularization. This analysis revealed that patients with a history of stroke exhibited significantly greater intensity of plaque enhancement versus the control group. This finding highlights the potential of CEUS as a valuable tool in distinguishing carotid plaques between stroke patients and non-stroke individuals. CEUS has the capability to detect neo-angiogenesis or enhanced inflammatory activity within the plaque of patients with IS. These processes are typically associated with increased plaque instability and rupture risk. Therefore, an increase in contrast enhancement intensity may serve as a potential biomarker of plaque vulnerability and tendency of stroke. Moreover, the rate of positive plaque enhancement was higher in IS patients versus non-stroke patients. The presence of enhanced contrast within the plaque, as detected by CEUS, further emphasizes the value of this modality in identifying high-risk atherosclerotic plaques. The existence of intra-plaque enhancement may indicate an active, unstable plaque state, which is associated with a higher risk of plaque rupture and consequently, an increased likelihood of stroke occurrence (Figure 5).

**Figure 6:**
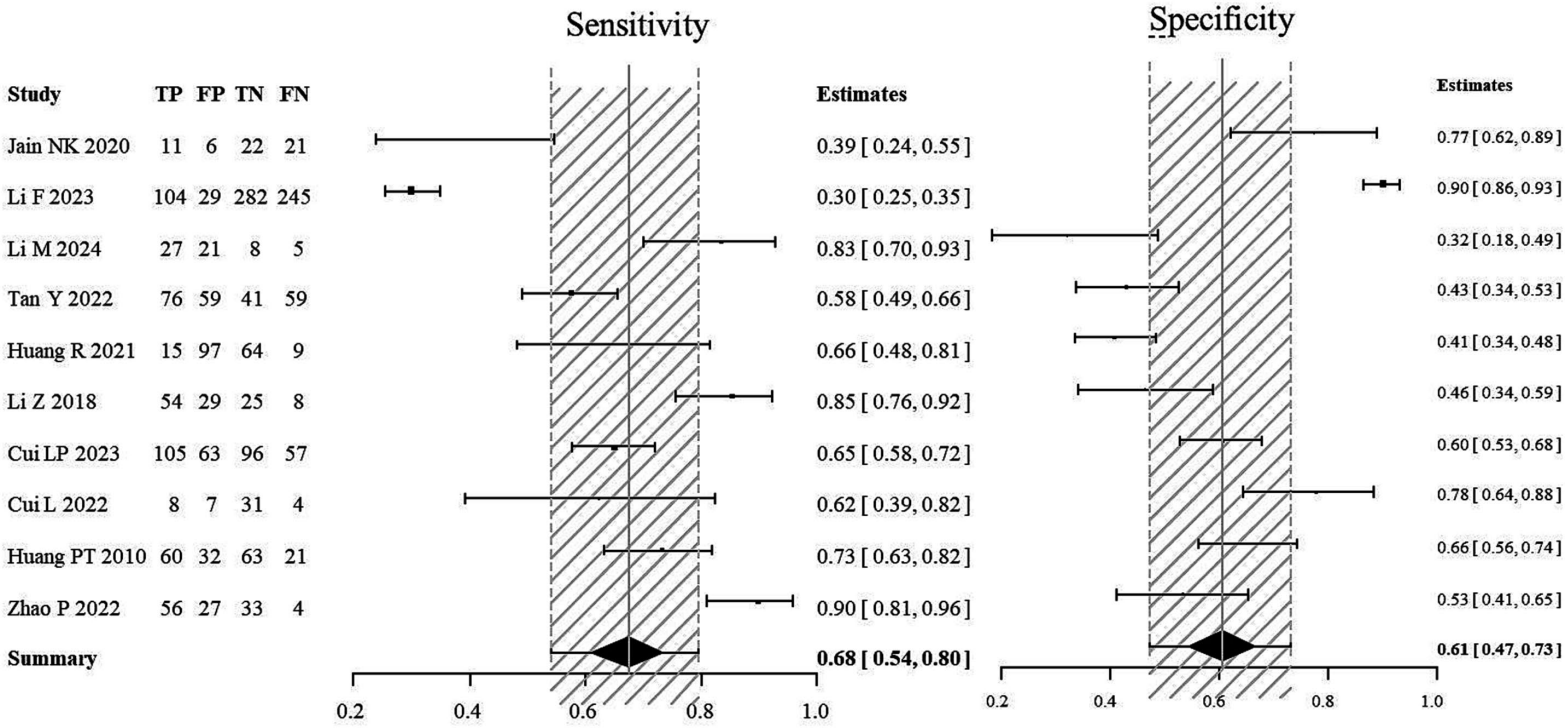
Funnel plot of publication bias. Observations from the funnel plots suggest the potential presence of publication bias for both outcome measures.

The sensitivity of CEUS in the diagnosis of IS indicates that it can correctly identify approximately 68% of patients who have experienced an IS event. While this value is not exceptionally high, it still maintains clinical utility, given the complex and multifactorial nature of stroke diagnosis. In non-IS patients, CEUS can correctly exclude approximately 61% of individuals. The relatively low specificity may reflect the limitations of CEUS in distinguishing plaque characteristics not associated with stroke, or the presence of similar plaque enhancement features in some non-stroke patients. The AUC was 0.697, indicating a moderate overall diagnostic performance of CEUS in differentiating IS patients from non-IS individuals. Although the AUC value did not reach a very high level, it still suggests the diagnostic potential of CEUS, particularly when combined with other clinical information and imaging modalities (Figure 6).

Vulnerable carotid plaques are a significant contributor to ischemic stroke, and the formation of new blood vessels (neovascularization) within the plaque plays a crucial role in increasing plaque vulnerability (15). The neomicrovasculature that develops within the plaque lacks the normal connective tissue support, making it prone to rupture and bleeding. This can lead to plaque instability and intraplaque hemorrhage (16). The presence of this neomicrovasculature within the carotid plaque serves as an independent risk factor for plaque rupture, hemorrhage, and a strong predictor of future cardiovascular and cerebrovascular events (17). The development of these delicate new blood vessels disrupts the structural integrity of the plaque, rendering it more unstable and prone to potentially devastating consequences, such as ischemic stroke.

CEUS, as a technique that reflects the microvascular system of the plaque, employs contrast agent microbubbles with similar characteristics to red blood cells, remaining within the vascular lumen (18). A number of studies have established a connection between the enhancement observed in CEUS and the histological vascular density of carotid plaques (19, 20). In patients undergoing carotid endarterectomy, pre-operative CEUS and CT examinations were conducted, followed by quantitative analysis of plaque enhancement and subsequent histopathological analysis. The results indicated a significantly higher CEUS plaque enhancement intensity in patients with CT-diagnosed IS compared to asymptomatic patients. Moreover, higher CEUS enhancement intensity correlated with a thinner fibrous cap and increased inflammatory infiltration on histopathology (21).

Quantitative analysis is an emerging trend in radiology, aiming to minimize subjectivity and enhance inter-observer consistency (22). The evaluation of neovascularization in atherosclerotic plaques using CEUS involves both semi-quantitative visual assessment and software-based quantitative assessment (23). The semi-quantitative assessment employs a scoring system proposed by Meng et al. (39), which categorizes plaque neovascularization into three levels: mild, with detectable microbubble flow only in the plaque’s adventitia; moderate, with detectable microbubble flow in the plaque shoulders and within the plaque; and severe, with detectable microbubble flow throughout the entire plaque, including the plaque tip. Quantitative assessment utilizes dedicated analysis software to quantify the plaque enhancement intensity and neovascularization within the plaque on CEUS. The contrast quantification software adjusts the ROI frame-by-frame based on plaque size and shape, and establishes another ROI in the center of the carotid lumen near the plaque’s proximal end as a reference (24). The software then automatically generates time-intensity curves for the plaque and lumen, calculating the enhancement intensity values and enhancement density (25). Schmidt et al. (26) confirmed a significant correlation between CEUS-detected neovascular quantity and histologically determined plaque vascular density, indicating good consistency between CEUS and histopathology in assessing plaque neovascularization. In patients scheduled for carotid endarterectomy, it is feasible to perform quantitative and volumetric imaging of the carotid artery and neovascularization within the plaque using CEUS (27). In this meta-analysis, both quantitative and semi-quantitative assessments supported the value of CEUS in ischemic stroke. The results of this meta-analysis underscore the potential role of CEUS in the assessment of carotid plaque stability and stroke risk. However, to establish CEUS as a routine clinical examination tool, further research is needed to optimize its diagnostic performance, determine the optimal examination protocol, and delineate its applicability across different patient populations. Additionally, exploring the combined use of CEUS with other imaging modalities, such as CT and MRI, may improve the accuracy and reliability of stroke risk assessment. While this meta-analysis provides new insights and evidence to support the application of CEUS in stroke prevention and management, future studies will need to address the current challenges and drive the further development of this technology.

However, this study has certain limitations. The included studies were primarily observational or cross-sectional in design, resulting in lower methodological quality. Additionally, the criteria for semi-quantitative plaque enhancement grading were not completely consistent, leading to unavoidable heterogeneity. Furthermore, the dichotomization of semi-quantitative plaque enhancement data may have reduced the interpretability of the results.

## 5 Conclusion

The CEUS characteristics of carotid plaques in IS significantly differ from those in patients without stroke, demonstrating higher plaque enhancement intensity and positive enhancement rate. These findings emphasize the prominent role of CEUS in the diagnosis and risk stratification of IS.

## Data Availability

The original contributions presented in the study are included in the article/supplementary material, further inquiries can be directed to the corresponding author/s.
